# Ryk modulates the niche activity of mesenchymal stromal cells by fine-tuning canonical Wnt signaling

**DOI:** 10.1038/s12276-020-0477-y

**Published:** 2020-07-28

**Authors:** Seon-Yeong Jeong, Jungmook Lyu, Jin-A Kim, Il-Hoan Oh

**Affiliations:** 1 Department of Medical Lifescience, The Catholic University of Korea, College of Medicine, Seoul, 137-701 Korea; 2 Myung-Gok Eye Research Institute, Department of Medical Science, Konyang University, Daejeon, 320-832 Korea; 3 Catholic High-Performance Cell Therapy Center, Department of Medical Lifescience, The Catholic University of Korea, College of Medicine, Seoul, 137-701 Korea

**Keywords:** Mesenchymal stem cells, Mesenchymal stem cells, Haematopoietic stem cells

## Abstract

The importance of modulating the intensity of Wnt signaling has been highlighted in various biological models, but their mechanisms remain unclear. In this study, we found that Ryk—an atypical Wnt receptor with a pseudokinase domain—has a Wnt-modulating effect in bone marrow stromal cells to control hematopoiesis-supporting activities. We first found that Ryk is predominantly expressed in the mesenchymal stromal cells (MSCs) of the bone marrow (BM) compared with hematopoietic cells. Downregulation of Ryk in MSCs decreased their clonogenic activity and ability to support self-renewing expansion of primitive hematopoietic progenitors (HPCs) in response to canonical Wnt ligands. In contrast, under high concentrations of Wnt, Ryk exerted suppressive effects on the transactivation of target genes and HPC-supporting effects in MSCs, thus fine-tuning the signaling intensity of Wnt in BM stromal cells. This ability of Ryk to modulate the HPC-supporting niche activity of MSCs was abrogated by induction of deletion mutants of Ryk lacking the intracellular domain or extracellular domain, indicating that the pseudokinase-containing intracellular domain mediates the Wnt-modulating effects in response to extracellular Wnt ligands. These findings indicate that the ability of the BM microenvironment to respond to extracellular signals and support hematopoiesis may be fine-tuned by Ryk via modulation of Wnt signaling intensity to coordinate hematopoietic activity.

## Hematology: Preserving a stable home for stem cells

Steady production of immune and blood cells depends on a signaling protein that helps maintain stable stem cell populations within the bone marrow. Hematopoietic stem cells (HSCs), which give rise to blood cells, reside within a supportive “niche” surrounded by mesenchymal stromal cells (MSCs), with extensive communication between the two populations. Researchers led by Il-Hoan Oh at The Catholic University of Korea, Seoul, have now identified a mechanism that MSCs employ to stabilize the niche environment through fine-tuning the signaling intensity of Wnt. Oh and colleagues focused on a signaling pathway that controls the undifferentiated state of HSCs, and showed that these signals are specifically modulated by an MSC protein known as Ryk. Without Ryk, MSCs can no longer promote HSC proliferation. However, when these signals are excessively strong, Ryk helps suppress proliferation to keep HSC numbers under control.

## Introduction

Hematopoietic stem cells (HSCs) are a rare subset of hematopoietic cells that can produce all blood cell lineages and regenerate bone marrow (BM) after transplantation into recipients^1,2^. The regenerative activity of HSCs is regulated by multidimensional mechanisms, including the microenvironment^3,4^. Accumulating studies have shown that the stem cell niche in the BM microenvironment plays a key regulatory role in the regeneration and maintenance of the hematopoietic system by controlling the self-renewal^5,6^ or quiescence^7–9^ of HSCs. The BM stem cell niche is comprised of various types of stromal cells, including those of mesenchymal and nonmesenchymal origin^10–12^. Specifically, mesenchymal stromal cells, including early-stage osteoblastic cells expressing Runx2^13^, nestin^14^ or leptin receptor^15^ or primitive (prx-1+) mesenchymal stromal cells (MSCs)^16^, have been identified as major mesenchymal niche cells in the BM. These MSCs serve as BM niche cells^17^ expressing crosstalk molecules, such as Jagged-1^6,18^, CXCL-12^16,19,20^, and angiopoietin-1^21^, that interact with HSCs to exert microenvironmental control of hematopoiesis.

Studies have shown that signals controlling the biological properties of MSCs in the BM microenvironment could have a regulatory effect on the regenerative activity of HSCs: adipogenic differentiation of MSCs in the BM suppresses HSC regeneration in myeloablated hosts^22^, whereas cells of the osteoblastic lineage support hematopoietic activity^5,6^. Accordingly, extensive studies are underway to identify the signals and molecular mechanisms that control the proliferation/self-renewal and differentiation of MSCs under various physiological and pathological conditions.

Wnts are secreted glycoproteins associated with the cell surface or extracellular matrix that influence various biological processes, including embryonic induction and cell fate specification. In the canonical Wnt signaling pathway, Wnt binds to seven-pass transmembrane Frizzled (Fz) family receptors and the single-pass coreceptors LRP 5 and 6 (LDL-receptor-related protein 5 and 6) to induce β-catenin stabilization. Subsequently, stabilized β-catenin translocates to the nucleus and forms a complex with the DNA-binding transcription factors TCF/LEF to activate a Wnt-controlled gene expression program^23^.

Notably, canonical Wnt signaling has been implicated in regulating the stromal activity of MSCs in the BM microenvironment. For example, we and others have recently shown that activation of Wnt/β-catenin signals in MSCs enhances HSC self-renewal by triggering crosstalk of Wnt-Notch signals in the stem cell niche^18,24^. Similarly, β-catenin expression in the BM stroma was necessary for the maintenance of long-term hematopoietic stem cells^18,25^. Moreover, different levels of β-catenin in MSCs have distinct effects on their cellular properties and on niche activity, supporting the self-renewal of HSCs^26^. These findings indicate that the intensity of canonical Wnt signals in MSCs should be fine-tuned for coordination of hematopoietic function, but the mechanisms for modulating this signal intensity remain unclear.

Related-to-receptor tyrosine kinase (Ryk) is a single pass transmembrane receptor for Wnt ligands. The cytoplasmic domain has a tyrosine kinase domain but lacks kinase activity^27–29^. Binding of Wnt to Ryk through their extracellular WIF (Wnt inhibitory factor-1 like) domain can initiate complex formation with Frz receptors for crosstalk in the Wnt signaling network^30^ to activate β-catenin-dependent and independent signaling pathways^29,31^. Although the tyrosine kinase domain in the intracellular domain of Ryk lacks enzymatic activity, it is cleaved by gamma-secretase and released for nuclear translocation^32^, suggesting that it may play an alternative role in regulating Wnt-related signals.

The biological functions of Ryk have been studied for their role during development, that is, the axonal outgrowth of neuronal tissues, cell migration and polarity^33^. Targeted disruption of Ryk causes various types of developmental defects in cardiovascular and craniofacial development with postnatal lethality^34,35^. In the hematopoietic system, Ryk was shown to exert a cell-autonomous effect on HSCs to control the proliferation and apoptosis of HSCs as well as their repopulating activities^36^. Similarly, Ryk was suggested as a cofactor for the noncanonical Wnt5a to control the Wnt5a-mediated quiescence of HSCs^37^ and thereby to control the response of HSCs to myeloablation injury^38^. However, despite these observations on the cell-autonomous effects on HSCs, the role of Ryk in the hematopoietic microenvironment controlling its niche activity remains unknown.

In this study, we showed that Ryk is predominantly expressed in the stromal cell compartment rather than the hematopoietic cells of the BM and that it controls the intensity of Wnt signaling in the mesenchymal stroma to modulate its responsiveness to the stimulatory effects from canonical Wnt signals.

## Materials and methods

### Cells

Murine bone marrow cells were isolated from the tibias and femurs of 8- to 10-week-old C57BL/6J-Ly5.1 (BL6) or C57BL/6J-Pep3b-Ly5.1 (Pep3b) mice by flushing the shaft with HF2 buffer (Hank’s Balanced Salt Solution (Welgene, Kyungsan-si,Korea) + 2% fetal bovine serum (HyClone, GE Healthcare, Logan, UT)) using a 3 ml syringe. Murine hematopoietic progenitors were enriched by pretreatment with 5-fluorouracil (150 mg/kg, 5-FU, Sigma Aldrich, St. Louis, MO) 4 days before BM harvest^39,40^. Mouse mesenchymal stromal cells (MSCs) from the BM were obtained by serial culture for adherent cells until all adherent cells became CD45 negative as described previously^18^ and were cultured in DMEM (HyClone) supplemented with 10% fetal bovine serum (HyClone), 2 mM l-glutamine (Gibco, Thermo Fisher Scientific, Waltham, MA), and antibiotic-antimycotic (Gibco).

### Plasmid construction

Nonspecific shRNA or *Ryk* shRNA in pFUGW, GFP alone, *Ryk*-WT, *Ryk*-dICD and *Ryk*-dECD in pFUIGW were previously described^30,32^. The murine *Ryk* targeting CRISPR/Cas9 system (pRGEN-mRyk-U6-sgRNA, pRGEN-cas9-CMV and reporter pHRS-mRyk-CMV) was purchased from ToolGen (Seoul, Korea).

### In vitro coculture

For coculture assays, mMSCs transduced with each lentiviral vector were irradiated with 15 Gy 1 day before coculture. Murine hematopoietic progenitors were cocultured with mMSCs for 5 days in MyeloCult M5300 (STEMCELL Technologies, Vancouver, BC, Canada) supplemented with antibiotic-antimycotic (Gibco), 100 ng/ml human Flt-3 ligand (FL, ProSpec-Tany TechnoGene, Ltd., Rehovort, Israel), 100 ng/ml mouse Stem Cell Factor (mSCF, ProSpec), 50 ng/ml human thrombopoietin (hTPO, ProSpec) and 10^−6^ M hydrocortisone (HC, STEMCELL Technologies). Wnt3a (10 ng/ml or 100 ng/ml, R&D Systems, Minneapolis, MN) or Wnt5a (10 ng/ml, R&D Systems) was added to the medium for coculturing, and BSA (Sigma Aldrich) was used to treat the control group. For colony forming assays of hematopoietic progenitors, hematopoietic cells were plated in methylcellulose media (MethoCult, STEMCELL Technologies) containing cytokines for 14 days and analyzed for colony numbers.

### Colony formation and osteogenic and adipogenic differentiation of mMSCs

Osteogenic and adipogenic differentiation of mMSCs was performed as previously described^41^, followed by Alizarin Red S (Sigma Aldrich) or Oil Red O (Sigma Aldrich) staining, respectively. The osteogenic mineralization an adipogenic lipid droplets were eluted and quantitatively measured by spectrophotometry at 550 and 520 nm, respectively. For colony formation, mMSCs transduced with each lentiviral vector were plated at a density of 50 cells per 60-mm dish. After incubation for 14 days, colonies were visualized by crystal violet (Sigma Aldrich) staining.

### TOP/FOP assay

The TOPFLASH and FOPFLASH reporter constructs containing eight TCF/LEF binding sites or mutated binding sites^42^ were transfected into cells. Luciferase activity was measured by the Luciferase Assay System (Promega, Madison, WI) according to the manufacturer’s instructions. Transfection efficiency was normalized to β-galactosidase activity.

### Western blotting and PCR

Western blots were performed using anti-actin antibody (clone C4; Millipore, Temecula, CA), mouse RYK antibody (R&D Systems), active β-catenin (Cell Signaling Technology, Danvers, MA) and β-catenin (Bethyl, Montgomery, TX), visualized with ECL (SuperSignal West Femto, Thermo Fisher Scientific) and quantified by LAS3000 software.

For RT-PCR analysis, RNA was purified from mMSCs and converted to cDNA using SuperScript III Reverse Transcriptase (Invitrogen, Thermo Fisher Scientific) and amplified using specific primers for m*Ryk* (5′-ATC CTA CCT TGC GGA TGA AG-3′, 5′-CAT CAT CGT CAC CTG AAC TT-3′), *Ryk*-ICD (5′-TCC AAG GTA CTT TTG GGC G-3′, 5′-TGT CCC CTA GGC AGT GGT AG-3′) and *Ryk*-ECD (5′-CAG TCA CTA CGC TCT GTC CT-3′, 5′-GCT CGA CCC GAA ACA CTG AT-3′). Real-time quantitative PCR (RQ-PCR) was performed with the Rotor-Gene 6000 system (Corbett Life Science, Australia) and SYBR Premix Ex Taq (TaKaRa, Japan). Relative levels of PCR products were determined after normalization to an endogenous *Gapdh* control.

### Flow cytometry

Cocultured cells were analyzed by staining with CD45.1 PE, CD45.2 PE, c-Kit APC (eBioscience, San Diego, CA), Sca-1 PE-Cy7, streptavidin APC-Cy7 (BD Pharmingen) and lineage cocktail biotin (a component of StemSep, STEMCELL Technologies). Labeled cells were analyzed using an LSRII flow cytometer (BD Biosciences).

## Results

### Ryk is preferentially expressed in the stromal compartment to modulate Wnt signaling

**Fig. 1:**
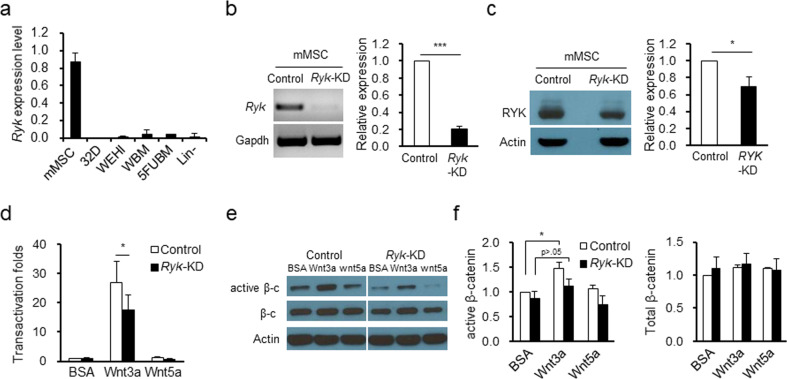
Ryk is preferentially expressed in the stromal compartment to modulate Wnt signaling. **a** Comparison of the expression of Ryk in hematopoietic and stromal cells. The expression levels of *Ryk* in each indicated cell type, as determined by RQ-PCR normalized to *Gapdh*, are shown. mMSC: murine mesenchymal stromal cells, WBM: whole bone marrow cells, 5FUBM: 5-FU bone marrow cells, Lin-: lineage depleted hematopoietic progenitor cells. **b**, **c** Knockdown of *Ryk* in mMSCs. mMSCs were infected with lentiviral vector harboring shRNA-*Ryk* (*Ryk*-KD) or shRNA-nonspecific (control), and a decrease in *Ryk* expression at the transcript level is shown by RT-PCR (left: representative gel profile, right: quantitative analysis), along with a decrease in the protein levels determined by Western blot profiles (left: image profile, right: quantitative analysis) (mean ± SEM, *p < 0.05, ***p < 0.001, *n* = 7 for RT-PCR, *n* = 3 for Western blot). **d** Effects of *Ryk*-KD on transactivation of Wnt target genes. *Ryk*-KD or control mMSCs were transfected with a luciferase reporter for Wnt-β-catenin target genes (TOPFLASH /FOPFLASH) to measure transactivation potential with normalization to β-galactosidase activity. The transactivation folds induced by Wnt3a or Wnt5a treatment in the control or *Ryk*-KD mMSCs were determined by the TOP/FOP ratio relative to that of the BSA-treated control (mean ± SEM, *p < 0.05, *n* = 4). **e**, **f** Effects of *Ryk*-KD on the accumulation of β-catenin. *Ryk*-KD or control mMSCs were treated with canonical (Wnt3a) or noncanonical (Wnt5a) ligands, and the levels of active (unphosphorylated) and total β-catenin were determined by Western blots and quantification by densitometry (mean ± SEM, *p < 0.05, *n* = 3).

Previous studies showed that the intensity of Wnt signaling on BM MSCs could be fine-tuned for coordinated control of the BM niche stimulating self-renewal of HSCs^43–45^. We thus investigated whether Ryk, an atypical Wnt receptor family member lacking tyrosine kinase, plays a role in modulating the signaling intensity of Wnt in BM MSCs. To study the potential role of Ryk in the hematopoietic system, we first examined the expression levels of Ryk in bone marrow (BM) hematopoietic tissue. *Ryk* expression was maintained at basal levels in hematopoietic cell lines, including 32D and WEHI cell lines^46^ (Fig. 1a). In primary bone marrow cells (BMCs), similar basal level expression was observed in whole BMCs and progenitor-enriched hematopoietic cells prepared by lineage depletion (Lin-) or by 5-FU treatment 4 days prior to harvest^39,40^. In contrast, markedly higher expression was observed in mesenchymal stromal cells (MSCs) obtained from BM. Thus, Ryk is predominantly localized in the stromal compartment rather than in the hematopoietic cells, suggesting a primary function in the BM microenvironment.

To examine the potential role in BM stroma, we next examined the role of Ryk in BM-derived MSCs by knocking down (KD) Ryk in these cells. Infection of MSCs with lentiviral vector encoding shRNA-*Ryk* resulted in significantly decreased levels of the *Ryk* transcript (Fig. 1b), with a less prominent decrease in protein levels (Fig. 1c).

Using these MSCs (MSCs/*Ryk*-KD), we first examined the effects on transactivation of Wnt target genes in response to Wnt ligands by using a reporter for TCF/LEF (TOP/FOP)^42^. *Ryk* KD did not affect the basal transactivation in MSCs cultured with BSA but significantly suppressed the Wnt3a-mediated transactivation of the reporter (Fig. 1d). This finding indicates that Ryk plays a role in modulating the MSC response to canonical Wnt signals. Supporting these findings, there was a significant increase in the accumulation of active (unphosphorylated) ß-catenin in response to Wnt3a stimulation in MSCs/sh-control that was lost in the *Ryk*-KD MSCs (Fig. 1e, f). The inhibitory role of *Ryk*-KD was specific to the canonical pathway of Wnt, since Wnt5a, the ligand for noncanonical Wnt signaling^27,28^, did not exhibit transactivation of TOP/FOP reporter genes, nor was their transactivation influenced by *Ryk*-KD (Fig. 1d, e).

**Fig. 2:**
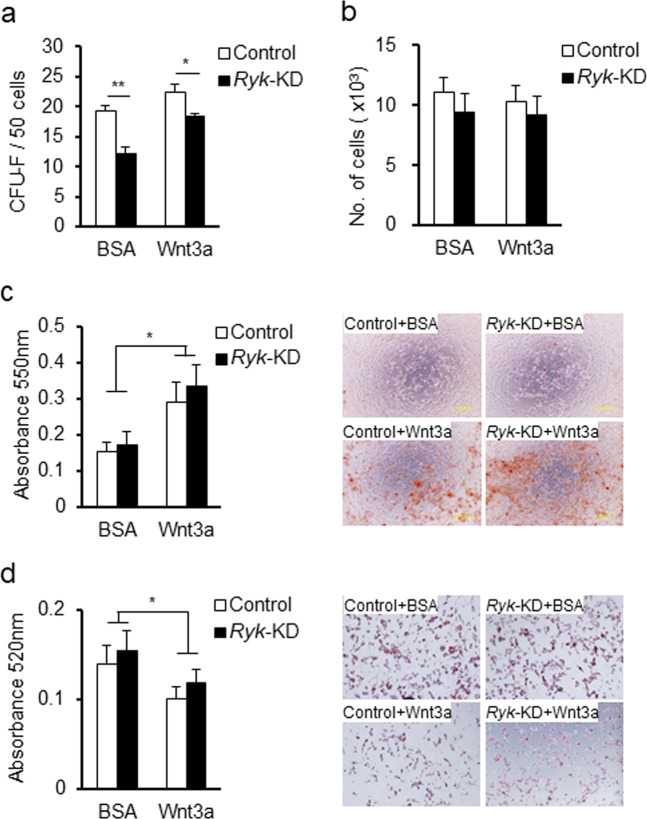
Effects of Ryk knockdown on the Wnt-responsive cellular function of mMSCs. **a** Effect of *Ryk*-KD on the frequency of mesenchymal progenitors in the mMSCs treated with Wnt3a. The numbers of colony forming unit-fibroblasts (CFU-F) from 50 plated cells are shown. **b** Effect of *Ryk*-KD on the proliferation of mMSCs. The number of cells in each indicated condition was measured 3 days after plating (2 × 10^3^ cells). **c**, **d** Effects of *Ryk*-KD on the differentiation of mMSCs. The control or *Ryk*-KD mMSCs were induced to differentiate into osteogenic or adipogenic lineages in the presence or absence of the Wnt3a ligand (10 ng/ml). The effect on osteogenic differentiation was measured by Alizarin Red S staining, as shown by representative images (right panel), and quantification by absorbance at 550 nm (left panel). Effects on adipogenic differentiation were measured by fat-droplet Oil Red O staining with images (right panel) and quantification by absorbance at 520 nm (left panel) (mean ± SEM, *p < 0.05, **p < 0.01, *n* = 3).

We next examined the effects of *Ryk*-KD on the cell autonomous functions of MSCs. Colony formation (CFU-F) of *MSCs/Ryk*-KD was decreased to modest levels compared to the MSCs/sh-control in the presence or absence of Wnt3a ligands (Fig. 2a), but there was no significant difference in their proliferation levels (Fig. 2b). These results suggest that Ryk might support the intrinsic clonogenic potential of MSCs without affecting the proliferation in culture. When MSC differentiation was examined, *Ryk*-KD had no effect on the Wnt3a-mediated increase in osteogenic differentiation and decrease in adipogenic differentiation (Fig. 2c, d). Together, these results show that Ryk modulates the intensity of canonical Wnt signals in MSCs but differentially influences various cell-autonomous functions of MSCs.

### Homozygotic loss of *Ryk* impairs colony formation of MSCs

**Fig. 3:**
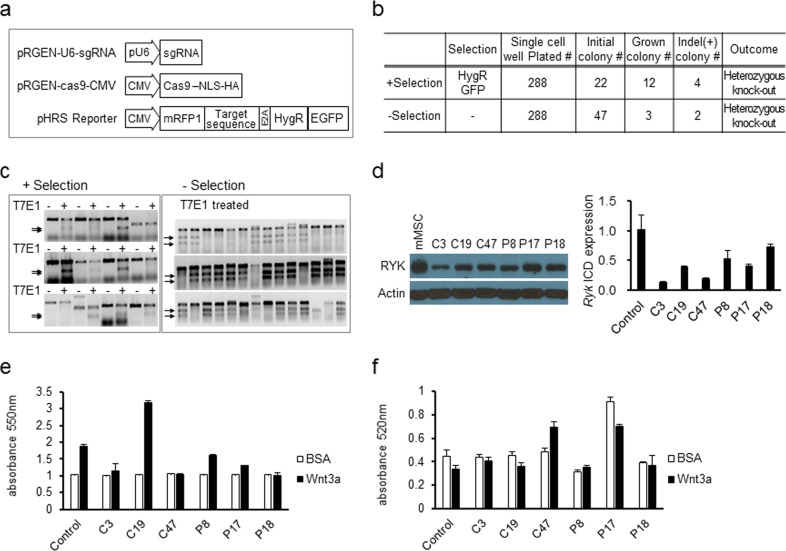
Effects of CRISPR-Cas9-mediated *Ryk* knockout on mMSC function. **a** Schematic structure of the CRISPR-Cas9 system. Single guide RNA (sgRNA) specific to murine *Ryk*, expression vectors for Cas9 and reporters for gene targeting assessed by hygromycin resistance (HygR) and enhanced green fluorescent protein (EGFP) are shown. **b** Outcomes of CRISPR-Cas9-mediated mutation on *Ryk* in mMSCs. The summary of the numbers of surviving cells and colonies of mMSCs from each indicated experimental stage for gene-targeting of *Ryk* by the CRISPR-Cas9 system is shown. The number of initial colonies is the number grown during the 1st passage of culture, and the number of grown colonies is the number that survived during passaging. **c** Representative profiles for indel formation assays during CRISPR-Cas9-mediated targeting of Ryk. The indel formation by T7E1 endonuclease treatment in the colonies that survived during culture with selection (EGFP + HygR) (left panel) and indel formations from mMSC colonies that exhibited initial growth in the absence of the selection (right panel) are shown. The indels are marked with an arrow. **d** Expression levels of Ryk in the mMSC colonies obtained after the gene-targeting procedure. The Western blot to measure the protein levels of RYK for each single-cell derived clone indicated (left panel) and the relative expression levels of the *Ryk* transcript determined by real-time quantitative PCR for the intracellular domain of *Ryk* in each indicated single cell-derived colony obtained from gene targeting (right panel) (*n* = 3) are shown. Each single colony was designated C3, C19, C14 (− selection group), P8, P17, and P18 (+ selection group). Note an approximately 50% decrease in the RYK protein and transcript levels in the gene-targeted single cell clones, indicating heterozygous *Ryk* mutations. **e** Effects of heterozygous *Ryk* knockout on the differentiation of different mMSCs in response to Wnt3a ligands. Each indicated *Ryk*-mutant MSCs was subjected to osteogenic or adipogenic differentiation in the presence or absence of Wnt3a ligands (10 ng/ml). The calorimetric determination of relative osteogenic differentiation at 550 nm (left panel) and adipogenic differentiation at 520 nm (right panel) compared to the control group (*n* = 3) is shown.

Next, to further examine the effects of *Ryk* deficiency in MSCs, we established MSC clones with stable loss of *Ryk* by CRISPR-Cas9-mediated gene knockout approaches^47^. MSCs were transfected with gene-targeting vectors along with the reporter for selection of gene-targeted clones, followed by single-cell sorting to obtain single-cell derived MSCs with disruption of *Ryk* (Fig. 3a). A total of 288 wells were plated with transfected single cells, but only 12 wells exhibited continued colony growth, four of which were positive for indel formation in the T7E1 assay due to mutation of the gene^48^ (Fig. 3b, c). However, these single clones that survived after selection were not full knockout mutants, as evidenced by only a partial decrease in the *Ryk* transcript and protein levels compared to those of the parental MSC population (Fig. 3d). This finding indicates that MSCs with a homozygous *Ryk* mutation did not form colonies during the selection procedure. As an alternative approach to obtain clones with homozygous mutations, we next screened the initially transfected MSCs by indel formation assays without selection by hygromycin resistance or green fluorescent protein expression. As shown, among the 288 wells with single-cell plating, initial colonies were found in 47 wells, but only two colonies with positive indel formation continued to grow (Fig. 3b). These were again colonies with heterozygous mutations exhibiting a similar decrease in transcript and protein levels as shown by selection screening (Fig. 3d). These findings suggest that MSCs with homozygous disruption of *Ryk* did not survive during clonal colonization, only producing colonies with heterozygous knockout. This result is consistent with our findings of decreased CFU-F colony numbers upon KD of *Ryk*.

Next, MSCs with heterozygous *Ryk* mutations were examined for the effect of Wnt3a during differentiation. There was no significant loss of response to Wnt3a during osteogenic and adipogenic differentiation in the heterozygous *Ryk* mutants compared to the WT, although clonal variations were observed (Fig. 3e, f), as with *Ryk*-KD. These findings reinforce our model that quantitative downregulation of *Ryk* in MSCs, while affecting their clonogenic potential, does not influence their response to Wnt ligands for multilineage differentiation of MSCs.

### Ryk modulates Wnt-induced activation of niche function in MSCs to control hematopoietic support

**Fig. 4:**
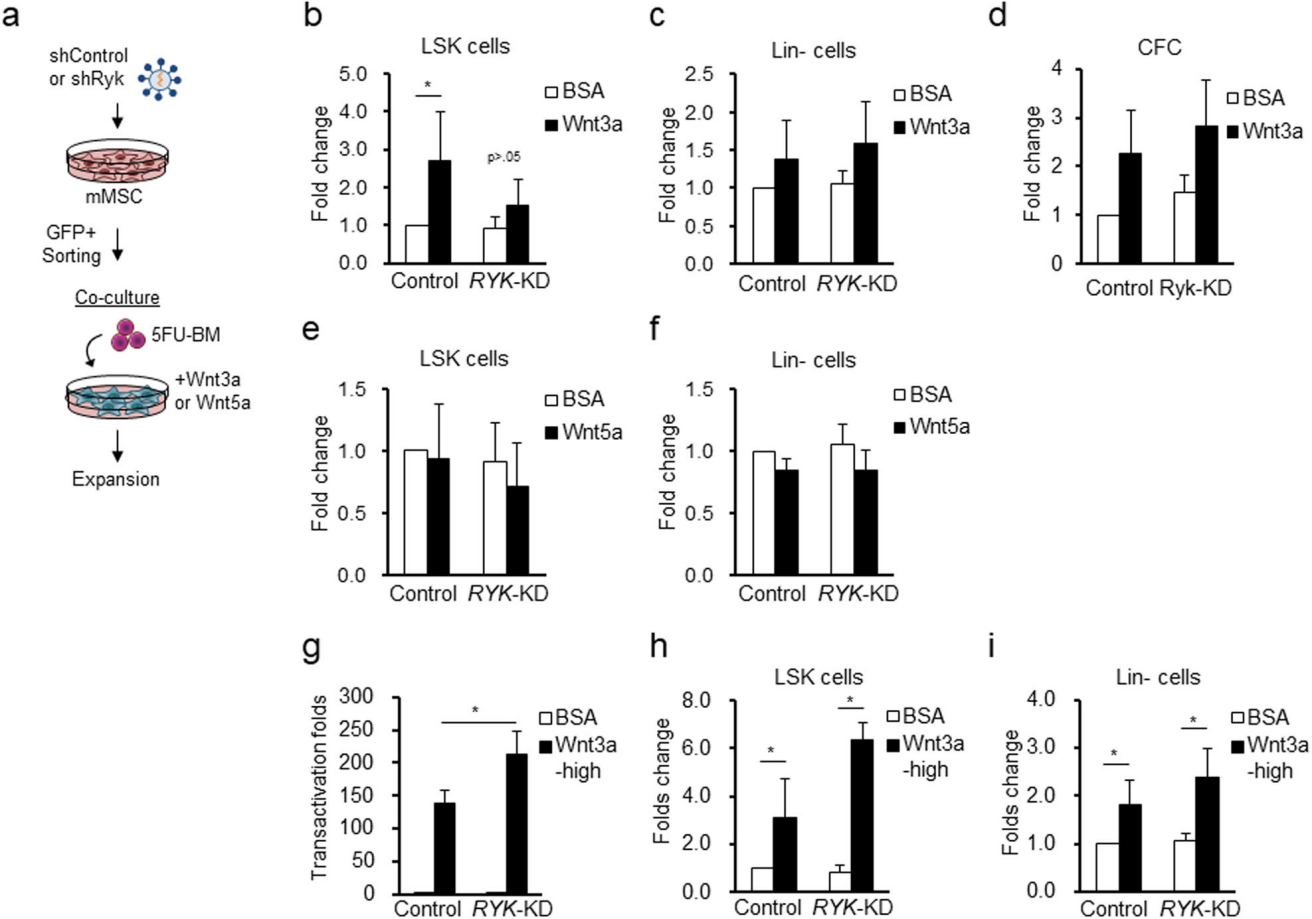
KD of *Ryk* selectively impairs canonical Wnt-mediated niche activation for primitive subsets of hematopoietic progenitors. **a** Experimental design. MSCs with *Ryk*-KD were cocultured with 5-FU-enriched hematopoietic progenitors for 5 days in the presence or absence of Wnt ligands (Wnt3a or Wnt5a) followed by measurement of expansion of each indicated subpopulation of hematopoietic cells. **b**–**d** Effects of *Ryk*-KD on canonical Wnt ligand (Wnt3a)-mediated activation of niche function. The relative fold expansion of LSK (Lin− Sca-1 + c-Kit+), lineage negative (Lin-) cells and colony forming cells (CFCs) relative to the coculture with control MSCs in the absence of Wnt ligands (mean ± SEM, *p < 0.05, *n* = 5) is shown. **e**, **f** Effects of *Ryk*-KD on niche activity of MSCs in response to noncanonical Wnt5a. The fold expansions of each indicated subpopulation of hematopoietic cells relative to the control group with BSA (mean ± SEM, *n* = 5) are shown. **g** Transactivation folds induced by high-dose Wnt3a (100 ng/ml) treatment in the control or *Ryk*-KD mMSCs were determined by the TOP/FOP ratio relative to the BSA-treated control (mean ± SEM, *p < 0.05, *n* = 4). **h**, **i** Effects of *Ryk*-KD on the niche activity of MSCs in response to high-dose canonical Wnt ligand (Wnt3a, 100 ng/ml). The relative fold expansion of LSK (**h**) and Lin- cells (**i**) relative to the coculture group with control MSCs (mean ± SEM, *p < 0.05, *n* = 4) is shown.

Since Ryk did not affect the Wnt response of MSCs in their control of cell-autonomous functions, we next examined the role of Ryk in the Wnt-mediated control of MSC niche activity supporting self-renewal of hematopoietic progenitors (HPCs)^17^. For this, we cocultured 5-FU-enriched HPCs with MSCs/*Ryk*-KD or control MSCs to compare the self-renewing expansion of primitive subsets of hematopoietic cells (LSK: Lin− Sca-1+ c-kit+) under each condition (Fig. 4a). In the absence of Wnt ligands (+BSA), there was no difference in the numbers of LSK cells after coculture with MSCs/*Ryk*-KD compared to those of the control MSC group (Fig. 4b). In contrast, in the presence of Wnt3a, the expansion of LSK observed with the control MSCs was abrogated upon coculture with MSC/*Ryk*-KD (Fig. 4b). Thus, Ryk controls the Wnt response of MSCs, stimulating HPC self-renewal.

However, the expansion of more downstream hematopoietic precursors, including Lin (−) hematopoietic cells and colony forming cells (CFCs), was not significantly affected by coculture with MSCs/*Ryk*-KD (Fig. 4c, d). This result indicates that their modulation of niche activity selectively affects primitive subsets rather than more mature hematopoietic precursors.

In contrast, the noncanonical ligand Wnt5a did not cause any effects on the HPC-supporting activity of MSCs for primitive (LSK) or more mature Lin (−) hematopoietic cells, and these effects were not influenced by KD of *Ryk* (Fig. 4e, f), indicating selective effects on canonical Wnt signaling.

Next, we also examined these Wnt-modulating effects of Ryk under higher concentrations of Wnt3a ligands. Surprisingly, in response to 10-fold higher concentrations of Wnt 3a, MSCs with *Ryk*-KD exhibited an increase in the transactivation of Wnt target genes (Fig. 4g), indicating that Ryk exerts a suppressive effect under excessively high amounts of Wnt3a ligands. Moreover, the HPC-supporting effects of MSCs were similarly increased in the MSCs with *Ryk*-KD as effects on the transactivation potential, which was again selective for the primitive population (LSK) without affecting the more downstream hematopoietic (Lin−) population (Fig. 4h, i).

Together, these results show that Ryk exerts modulatory effects on Wnt signaling in a dose-dependent manner that supports low-level signaling but suppresses excessively high Wnt signaling, suggesting fine-tuning of the Wnt responses of MSCs and their HPC-stimulating effects.

### Ligand-activated intracellular domain of Ryk participates in the Wnt-modulating effects of Ryk in MSCs

**Fig. 5:**
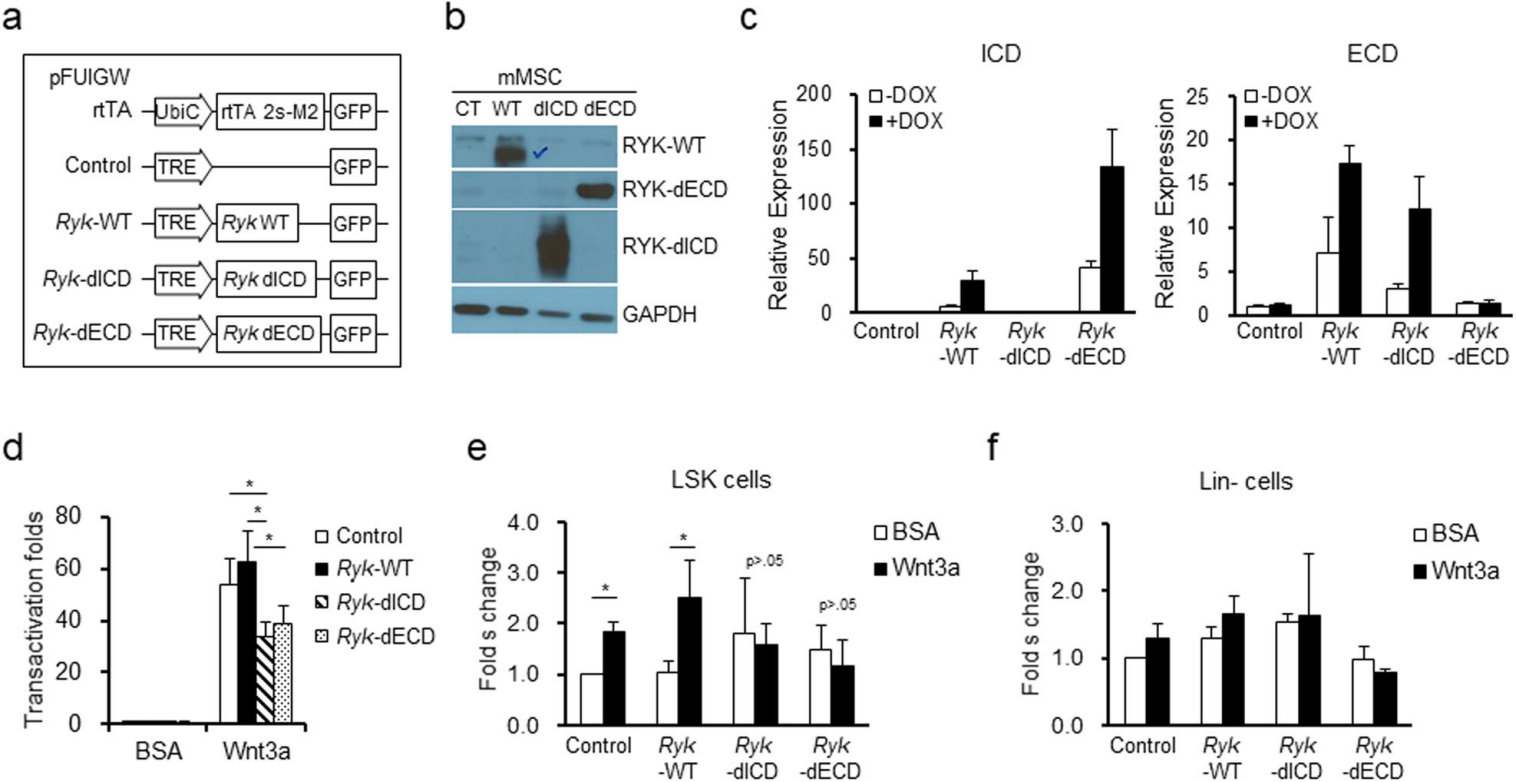
The niche-modulating effect is dependent on the intracellular domain of Ryk. **a** Schematic structures of vectors for doxycycline-inducible expression of WT and the mutants with a deletion of the intracellular domain (dICD) or extracellular domain (dECD) of Ryk. **b** Validation of transgenic expression of *Ryk* and its mutants in MSCs. MSCs transfected with each construct were doxycycline induced and sort-purified by GFP and examined for protein products of the construct. The Western blot analysis for WT and mutants (*Ryk*-dICD, *Ryk*-dECD) detected by an antibody against RYK is shown. **c** Validation of expression by qRT-PCR. The fold expression of each construct in the presence or absence of doxycycline (1 µg/ml) detected by PCR primers specific to the intracellular domain (ICD) (left) or extracellular domain (ECD) (right) of *Ryk* is shown. **d** Effect on transactivation of Wnt target genes. The transactivation folds of each indicated construct in MSCs normalized to the BSA-treated control group (mean ± SEM, *p < 0.05, *n* = 3) are shown. **e**, **f** Effects on HPC supporting activity. Each group of MSCs was cocultured with HPCs for 5 days to compare their niche activity supporting the self-renewing expansion of HPCs in the presence or absence of Wnt3a. The fold changes in the numbers of primitive HPC subsets (LSK) and lineage negative (Lin−) populations are shown (mean ± SEM, *p < 0.05, *n* = 3).

Since Ryk has Wnt ligand binding activity through a WIF motif in its extracellular domain (ECD), the Wnt-modulatory effects of Ryk on MSC niche function could be attributed to their inhibition of Wnt ligands by competing with Frz receptors. Alternatively, the modulating effects of Ryk could be an intrinsic function of Ryk as a receptor for Wnt ligands, transmitting modulatory signals through their intracellular domain (ICD). To discriminate between these possibilities, we next examined the effects of a mutant form of Ryk (*Ryk*-dICD), where the ICD of Ryk is deleted while the ECD and transmembrane domain are preserved^32^ (Fig. 5a). Thus, lentiviral vectors encoding *Ryk*-WT or *Ryk*-dICD under a doxycycline-inducible promoter were constructed and transduced into MSCs. The inducible expression of these transgenes was confirmed by a marked increase in both the transcript and protein levels upon doxycycline treatment (Fig. 5b, c). The transactivation of Wnt-target genes (TOP/FOP reporters) by Wnt3a stimulation was not affected by overexpression of *Ryk*-WT but was significantly decreased by induction of *Ryk*-dICD (Fig. 5d), indicating that the ICD of Ryk is necessary for the optimal intensity of Wnt3a signals.

We also examined the effect of Wnt on MSCs in their support for HPC self-renewal. Overexpression of *Ryk*-WT in MSCs did not affect the Wnt3a-mediated increase in self-renewing expansion of LSK cells. In contrast, induction of *Ryk*-dICD in MSCs abrogated the enhancing effects, and no increase in the expansion of LSK cells was observed (Fig. 5e). In contrast, no significant difference was observed for the expansion of more downstream Lin (−) hematopoietic cells, reinforcing the selective effects of Ryk on primitive hematopoietic subsets (Fig. 5f). These results further showed that Ryk serves as a modulator of Wnt signaling intensity, controlling the HPC-supporting activity of MSCs in a manner dependent on the ICD of Ryk, rather than just squelching of Wnt ligands by Wnt-binding ECD.

Having observed the role of ICD in Ryk, we next examined whether ICD can exert independent effects to support the Wnt effects without ligand binding of ECD. For this, we overexpressed another mutant of Ryk lacking the extracellular domain (dECD) in MSCs. The mutant *Ryk* lacking the ECD similarly decreased the transactivation of Wnt3a target genes (TOP/FOP) (Fig. 5d) and similarly abrogated the Wnt3a-mediated HPC-stimulating effects of MSCs (Fig. 5e). These findings indicate that orphan ICD without ECD-ligand interactions similarly impairs the function of Ryk in modulating the optimal intensity of Wnt signaling.

Together, these results show that Ryk may exert its Wnt-modulating effects through pseudokinase-containing ICD in a manner dependent on the interaction of Wnt ligands and ECD to control the self-renewal of the primitive hematopoietic pool.

## Discussion

The microenvironment of the BM plays a critical role in controlling the regenerative capacity of HSCs, responding to extrinsic signals to accommodate the physiological need for hematopoiesis with reversible switching between dormancy and self-renewal of HSCs^49–51^. Accordingly, fine-tuning this niche activity to control the stimulatory effects of the mesenchymal niche on HSC self-renewal is critical for balancing maintenance of the HSC reservoir in BM with the activation of hematopoietic output. However, the signaling network that can modulate the niche activity of MSCs in response to regenerative stimuli has not yet been fully characterized.

In this light, recent studies showed that MSCs exert different levels of HSC-stimulating effects depending on their cellular heterogeneity related to aging^52^, the difference in the molecular gradients for epithelial-mesenchymal transition^53^, or culture conditions^54^, and MSCs can undergo adaptive changes during physiological stimulation for HSC regeneration^40^, indicating a dynamic control of niche activity with respect to the physiological signals to control hematopoiesis.

We previously showed that canonical Wnt signals target mesenchymal cells in the BM stem cell niche to stimulate HSC self-renewal in a contact-dependent manner, controlling cell fate decisions for HSCs^24,55^. These findings were similarly observed through functional changes of stromal cells in response to Wnt stimulation, which led to the enhancement of HSC self-renewal^56^.

Of note, accumulating studies have revealed dose-dependent responses of Wnt signaling in various types of cells, indicating the importance of fine-tuning Wnt signaling intensity for optimal regulation of Wnt response in the cells^43–45^. Consistent with these dose-dependent responses, the difference in β-catenin levels accumulating in MSCs was shown to distinctively regulate the behavior of MSCs, causing a difference in their HSC-stimulating effects^26^. However, it is not clear how the intensity of Wnt signaling in cells could be modulated in the physiological microenvironment of BM stromal cells.

In this study, we examined the possibility that the intensity of Wnt signaling in mesenchymal cells—the physiological stimuli for niche activation—is modulated by another Wnt interacting receptor, Ryk, to fine-tune niche activity for homeostatic control of hematopoietic activity.

Previous studies have shown a role for Ryk in hematopoietic cells via direct effects on HSCs controlling their proliferation, quiescence, and apoptosis^36–38^, suggesting a role of Ryk in hematopoietic cells. However, we proposed an alternative role for Ryk in the stromal component of the BM, based on our findings that *Ryk* expression is predominantly observed in the mesenchymal stroma of the BM rather than the hematopoietic cells (Fig. 1a), which indicated their functional role in the stromal microenvironment in the physiological annotations of Wnt signaling. Consistent with this possibility, previous findings showed that Frz receptors for Wnt signaling are predominantly enriched in stromal cells compared with hematopoietic cells in the BM microenvironment, and the HSC-stimulating effects of Wnt signals are activated when they target the stromal compartment rather than hematopoietic cells^18^.

Unexpectedly, we found no significant effects of downregulating Ryk in MSCs on their cell-autonomous functions, including proliferation and differentiation into osteogenic/adipogenic cells. While CFU-F was decreased in MSCs by downregulation of Ryk, the decrease was not dependent on Wnt signaling (Fig. 2a), suggesting that the colony forming activity of MSCs is related to an intrinsic function of Ryk. However, the lack of effect on these cell-autonomous functions was not due to lack of Ryk signaling activity in MSCs, since MSCs with *Ryk*-KD exhibited a significant decrease in the TOP/FOP transactivation assay as well as a decrease in the protein levels of active β-catenin (unphosphorylated form) (Fig. 1d, e).

These findings were similarly reproduced in the CRISPR-Cas9-mediated gene-targeting model, which exhibited a similar lack of Wnt effect on cell autonomous functions during differentiation (Fig. 3e, f). However, we failed to retrieve clones with homozygous loss of *Ryk*, producing only heterozygous clones (Fig. 3b, d). This finding suggests that Ryk also has a Wnt-independent role in the maintenance of colony forming cell populations of MSCs, as recently inferred by similar effects on the stemness control of cancer cell lines^57^. However, further studies are necessary to confirm the possibility that Ryk is involved in the development of clonogenic subsets of MSCs.

In contrast to the lack of Wnt response in cell-autonomous functions of MSCs, downregulation of Ryk significantly reduced the stimulatory effect of Wnt3a on the niche activity of MSCs to support HPC self-renewal (Fig. 4b). Importantly, these modulating effects of Ryk on Wnt signaling were dependent on the dose of Wnt 3a ligands, i.e., Ryk supported low Wnt signaling but suppressed high Wnt signaling, thus modulating the intensity of Wnt signaling in MSCs (Fig. 4g, h).

Together, these findings suggested that Ryk-mediated modulation of Wnt signaling is physiologically relevant for the MSC niche activity, rather than the regulation of cell-autonomous functions of MSCs, modulating Wnt signaling in MSCs and, thereby, fine-tuning HPC self-renewal in the hematopoietic microenvironment.

Of note, the niche-modulating effects of Ryk were observed selectively on the primitive subsets (LSK) of hematopoietic progenitors but not on more mature populations. Consistent with these selective effects on primitive subsets of hematopoietic cells, we previously showed similar selectivity on primitive subsets in Wnt-activated stromal cells, where self-renewal of HSCs was selectively promoted without influencing the committed hematopoietic cell population^18^. These findings suggest that the modulatory effects of Ryk on Wnt activation of the mesenchymal niche are functionally relevant to the maintenance of HSC pools in the BM for the homeostatic control of blood cell production.

Interestingly, the role of Ryk on the stromal niche activity of MSCs was observed only upon Wnt3a stimulation and not Wnt5a stimulation (Fig. 4e). In contrast, previous studies showed direct effects of Ryk on HPCs in a Wnt5a-dependent manner^36–38^, indicating distinct functions of Wnt family ligands in the BM microenvironment in the context of their cellular targets. Notably, noncanonical Wnt5a, secreted by stromal cells, was shown to promote HSC self-renewal and repopulation by directly stimulating HSCs^58–61^. In contrast, canonical Wnt signals, when directly activated in HSCs, caused loss of HSCs and defective hematopoiesis, unlike their stimulatory effects on HSC self-renewal when targeted to the stromal compartment of BM^18,62,63^. Integrating these findings, it is possible that the physiological roles of Wnt signals are compartmentalized in the BM microenvironment, wherein the effects on HSCs are mediated by noncanonical Wnt signals, whereas the effects on stromal niche activity are mediated through canonical Wnt signals. Taken together, these findings suggest that Ryk should serve as a modulator of canonical Wnt signaling in stromal cells in BM but also function as a modulator of noncanonical Wnt5a signals in hematopoietic progenitors.

Of note, considering that the kinase domain in the ICD is inactive in Ryk, it is possible that the modulatory effects of Ryk were mediated through their squelching of Wnt ligands by competitive binding. However, we found that overexpression of *Ryk*-WT had no effect on the cell-autonomous functions of MSCs or their HSC-supporting effects (Fig. 5e), precluding ligand squelching as a possible mechanism. Instead, inducible expression of *Ryk*-dICD in MSCs reproduced the effects caused by downregulation of Ryk (Fig. 5e), i.e., similar abrogation of the Wnt3a-mediated enhancement of niche activity of MSCs supporting HSC self-renewal. These findings indicate that the intracellular domain of Ryk, despite the lack of tyrosine kinase activity, plays a role in the modulation of Wnt3a signaling activity as a functional entity of the receptors. In support of this possibility, it was shown that the tyrosine kinase domain in the ICD of Ryk is cleaved during signal transmission^32^, indicating that the ICD mediates Ryk functions via mechanisms other than enzymatic activity. However, another mutant of Ryk with deletion of the ECD caused a similar loss of transcription and HSC-supporting activity in Wnt 3a-mediated stimulatory effects, indicating that the optimal activity of ICD to support Wnt signals requires a functional interaction of ECD with their ligand. These findings together reinforce the view that Ryk should play a role in modulating Wnt signaling intensity in the BM microenvironment through the ICD in a manner dependent on the extracellular interaction with Wnt ligands.

The importance of modulating the signaling intensity of Wnt has been highlighted with findings for the dose dependence of the Wnt signaling response in various tissues^45^. Accumulating studies have shown that the strength of Wnt signaling should be fined-tuned for optimal function of HSCs^43,44^^,^ and its dysregulation has been implicated in various types of leukemic cell development and resistance to therapy^44,64–66^. Similarly, dysregulated Wnt signals were observed in the BM stromal cells of leukemia patients^67,68^, whereas lack of canonical Wnt signaling in BM stroma led to deficiency of HSC repopulating activities^69–71^. Furthermore, altered signaling intensity of Wnt was shown to be associated with aging of the BM niche and hematopoietic cells^72–74^. Accordingly, fine-tuning of Wnt signaling intensity is crucial for homeostatic control of normal hematopoietic cells during their regeneration and aging. In this light, our findings for dependency of Wnt signaling on the functional expression of Ryk suggest that changes in the expression or activity of Ryk in BM could constitute another factor orchestrating Wnt signaling in the BM microenvironment for homoeostasis of the hematopoietic system. Further studies are warranted to understand the physiological regulation of hematopoiesis by signaling from Ryk and their roles under disease conditions.

In summary, our data demonstrated that Ryk is compartmentalized in the BM stroma as a Wnt receptor and that it modulates the signaling intensity of Wnt in the BM microenvironment to optimize cellular responses to physiological stimuli mediated by Wnt signals for fine-tuning hematopoietic outputs.

## References

[1] Mendelson A, Frenette PS (2014). Hematopoietic stem cell niche maintenance during homeostasis and regeneration. Nat. Med..

[2] Morrison SJ, Scadden DT (2014). The bone marrow niche for haematopoietic stem cells. Nature.

[3] Oh IH, Humphries RK (2012). Concise review: multidimensional regulation of the hematopoietic stem cell state. Stem Cells.

[4] Oh IH, Kwon KR (2010). Concise review: multiple niches for hematopoietic stem cell regulations. Stem Cells.

[5] Zhang J (2003). Identification of the haematopoietic stem cell niche and control of the niche size. Nature.

[6] Calvi LM (2003). Osteoblastic cells regulate the haematopoietic stem cell niche. Nature.

[7] Stier S (2005). Osteopontin is a hematopoietic stem cell niche component that negatively regulates stem cell pool size. J. Exp. Med..

[8] Nilsson SK (2005). Osteopontin, a key component of the hematopoietic stem cell niche and regulator of primitive hematopoietic progenitor cells. Blood.

[9] Arai F, Suda T (2007). Maintenance of quiescent hematopoietic stem cells in the osteoblastic niche. Ann. N. Y Acad. Sci..

[10] Hooper AT (2009). Engraftment and reconstitution of hematopoiesis is dependent on VEGFR2-mediated regeneration of sinusoidal endothelial cells. Cell Stem Cell.

[11] Mendez-Ferrer S, Lucas D, Battista M, Frenette PS (2008). Haematopoietic stem cell release is regulated by circadian oscillations. Nature.

[12] Spiegel A (2007). Catecholaminergic neurotransmitters regulate migration and repopulation of immature human CD34+ cells through Wnt signaling. Nat. Immunol..

[13] Chitteti BR (2010). Osteoblast lineage cells expressing high levels of Runx2 enhance hematopoietic progenitor cell proliferation and function. J. Cell Biochem..

[14] Mendez-Ferrer S (2010). Mesenchymal and haematopoietic stem cells form a unique bone marrow niche. Nature.

[15] Ding L, Saunders TL, Enikolopov G, Morrison SJ (2012). Endothelial and perivascular cells maintain haematopoietic stem cells. Nature.

[16] Greenbaum A (2013). CXCL12 in early mesenchymal progenitors is required for haematopoietic stem-cell maintenance. Nature.

[17] Kfoury Y, Scadden DT (2015). Mesenchymal cell contributions to the stem cell niche. Cell Stem Cell.

[18] Kim JA (2009). Identification of a stroma-mediated Wnt/beta-catenin signal promoting self-renewal of hematopoietic stem cells in the stem cell niche. Stem Cells.

[19] Omatsu Y (2010). The essential functions of adipo-osteogenic progenitors as the hematopoietic stem and progenitor cell niche. Immunity.

[20] Sugiyama T, Kohara H, Noda M, Nagasawa T (2006). Maintenance of the hematopoietic stem cell pool by CXCL12-CXCR4 chemokine signaling in bone marrow stromal cell niches. Immunity.

[21] Nakamura Y (2007). Angiopoietin-1 supports induction of hematopoietic activity in human CD34- bone marrow cells. Exp. Hematol..

[22] Naveiras O (2009). Bone-marrow adipocytes as negative regulators of the haematopoietic microenvironment. Nature.

[23] Kikuchi A, Kishida S, Yamamoto H (2006). Regulation of Wnt signaling by protein-protein interaction and post-translational modifications. Exp. Mol. Med..

[24] Oh IH (2010). Microenvironmental targeting of Wnt/beta-catenin signals for hematopoietic stem cell regulation. Expert Opin. Biol. Ther..

[25] Nemeth MJ, Mak KK, Yang Y, Bodine D (2009). M. beta-Catenin expression in the bone marrow microenvironment is required for long-term maintenance of primitive hematopoietic cells. Stem Cells.

[26] Kim JA, Choi HK, Kim TM, Leem SH, Oh IH (2015). Regulation of mesenchymal stromal cells through fine tuning of canonical Wnt signaling. Stem cell Res..

[27] Mikels AJ, Nusse R (2006). Purified Wnt5a protein activates or inhibits beta-catenin-TCF signaling depending on receptor context. PLoS Biol..

[28] Wu CH, Nusse R (2002). Ligand receptor interactions in the Wnt signaling pathway in Drosophila. J. Biol. Chem..

[29] Roy JP, Halford MM, Stacker SA (2018). The biochemistry, signalling and disease relevance of RYK and other WNT-binding receptor tyrosine kinases. Growth Factors.

[30] Lu W, Yamamoto V, Ortega B, Baltimore D (2004). Mammalian Ryk is a Wnt coreceptor required for stimulation of neurite outgrowth. Cell.

[31] Anastas JN (2015). Functional crosstalk between WNT signaling and Tyrosine Kinase signaling in cancer. Semin. Oncol..

[32] Lyu J, Yamamoto V, Lu W (2008). Cleavage of the Wnt receptor Ryk regulates neuronal differentiation during cortical neurogenesis. Dev. Cell.

[33] Blakely BD (2013). Ryk, a receptor regulating Wnt5a-mediated neurogenesis and axon morphogenesis of ventral midbrain dopaminergic neurons. Stem Cells Dev..

[34] Kugathasan K (2018). Deficiency of the Wnt receptor Ryk causes multiple cardiac and outflow tract defects. Growth Factors.

[35] Halford MM (2000). Ryk-deficient mice exhibit craniofacial defects associated with perturbed Eph receptor crosstalk. Nat. Genet..

[36] Famili F (2016). The non-canonical Wnt receptor Ryk regulates hematopoietic stem cell repopulation in part by controlling proliferation and apoptosis. Cell Death Dis..

[37] Povinelli BJ, Nemeth MJ (2014). Wnt5a regulates hematopoietic stem cell proliferation and repopulation through the Ryk receptor. Stem Cells.

[38] Povinelli BJ, Srivastava P, Nemeth MJ (2015). Related-to-receptor tyrosine kinase receptor regulates hematopoietic stem and progenitor sensitivity to myelosuppressive injury in mice. Exp. Hematol..

[39] Weissman TDRaIL (1997). Phenotypic and functional changes induced at the clonal level in hematopoietic stem cells after 5-fluorouracil treatment. Blood.

[40] Jeong SY, Kim JA, Oh IH (2018). The adaptive remodeling of stem cell niche in stimulated bone marrow counteracts the leukemic Niche. Stem cells (Dayt., Ohio).

[41] Jung J (2009). Mesenchymal stromal cells expanded in human allogenic cord blood serum display higher self-renewal and enhanced osteogenic potential. Stem Cells Dev..

[42] Biechele TL, Moon RT (2008). Assaying beta-catenin/TCF transcription with beta-catenin/TCF transcription-based reporter constructs. Methods Mol. Biol..

[43] Famili F (2016). High levels of canonical Wnt signaling lead to loss of stemness and increased differentiation in hematopoietic stem cells. Stem Cell Rep..

[44] Luis TC, Ichii M, Brugman MH, Kincade P, Staal FJ (2012). Wnt signaling strength regulates normal hematopoiesis and its deregulation is involved in leukemia development. Leukemia.

[45] Luis TC (2011). Canonical wnt signaling regulates hematopoiesis in a dosage-dependent fashion. Cell Stem Cell.

[46] Simoneaux DK (1995). The receptor tyrosine kinase-related gene (ryk) demonstrates lineage and stage-specific expression in hematopoietic cells. J. Immunol..

[47] Kim JM, Kim D, Kim S, Kim JS (2014). Genotyping with CRISPR-Cas-derived RNA-guided endonucleases. Nat. Commun..

[48] Mashal RD, Koontz J, Sklar J (1995). Detection of mutations by cleavage of DNA heteroduplexes with bacteriophage resolvases. Nat. Genet..

[49] Wilson A (2008). Hematopoietic stem cells reversibly switch from dormancy to self-renewal during homeostasis and repair. Cell.

[50] Essers MA (2009). IFNalpha activates dormant haematopoietic stem cells in vivo. Nature.

[51] Trumpp A, Essers M, Wilson A (2010). Awakening dormant haematopoietic stem cells. Nat. Rev. Immunol..

[52] Lee GY, Jeong SY, Lee HR, Oh IH (2019). Age-related differences in the bone marrow stem cell niche generate specialized microenvironments for the distinct regulation of normal hematopoietic and leukemia stem cells. Sci. Rep..

[53] Jeon S (2017). Shift of EMT gradient in 3D spheroid MSCs for activation of mesenchymal niche function. Sci. Rep..

[54] Kim JH (2016). Heterogeneous Niche activity of ex-vivo expanded MSCs as factor for variable outcomes in hematopoietic recovery. PloS ONE.

[55] Kim JA (2015). Microenvironmental remodeling as a parameter and prognostic factor of heterogeneous leukemogenesis in acute myelogenous leukemia. Cancer Res..

[56] Ichii M, Frank MB, Iozzo RV, Kincade PW (2012). The canonical Wnt pathway shapes niches supportive of hematopoietic stem/progenitor cells. Blood.

[57] Adamo A (2017). RYK promotes the stemness of glioblastoma cells via the WNT/ beta-catenin pathway. Oncotarget.

[58] Buckley SM (2011). Maintenance of HSC by Wnt5a secreting AGM-derived stromal cell line. Exp. Hematol..

[59] Murdoch B (2003). Wnt-5A augments repopulating capacity and primitive hematopoietic development of human blood stem cells in vivo. Proc. Natl Acad. Sci. USA.

[60] Nemeth MJ, Topol L, Anderson SM, Yang Y, Bodine DM (2007). Wnt5a inhibits canonical Wnt signaling in hematopoietic stem cells and enhances repopulation. Proc. Natl Acad. Sci. USA.

[61] Schreck C (2017). Niche WNT5A regulates the actin cytoskeleton during regeneration of hematopoietic stem cells. J. Exp. Med..

[62] Kirstetter P, Anderson K, Porse BT, Jacobsen SE, Nerlov C (2006). Activation of the canonical Wnt pathway leads to loss of hematopoietic stem cell repopulation and multilineage differentiation block. Nat. Immunol..

[63] Scheller M (2006). Hematopoietic stem cell and multilineage defects generated by constitutive beta-catenin activation. Nat. Immunol..

[64] Ahmadzadeh A, Norozi F, Shahrabi S, Shahjahani M, Saki N (2016). Wnt/beta-catenin signaling in bone marrow niche. Cell Tissue Res..

[65] Arrigoni E (2018). Concise review: chronic myeloid leukemia: stem cell niche and response to pharmacologic treatment. Stem Cells Transl. Med..

[66] Zhou HS, Carter BZ, Andreeff M (2016). Bone marrow niche-mediated survival of leukemia stem cells in acute myeloid leukemia: Yin and Yang. Cancer Biol. Med..

[67] Azevedo PL (2019). Canonical WNT signaling pathway is altered in mesenchymal stromal cells from acute myeloid leukemia patients and is implicated in BMP4 down-regulation. Transl. Oncol..

[68] Chandran P (2015). Mesenchymal stromal cells from patients with acute myeloid leukemia have altered capacity to expand differentiated hematopoietic progenitors. Leuk. Res..

[69] Stoddart A (2017). Inhibition of WNT signaling in the bone marrow niche prevents the development of MDS in the Apc(del/+) MDS mouse model. Blood.

[70] Fleming HE (2008). Wnt signaling in the niche enforces hematopoietic stem cell quiescence and is necessary to preserve self-renewal in vivo. Cell Stem Cell.

[71] Lane SW (2010). The Apc(min) mouse has altered hematopoietic stem cell function and provides a model for MPD/MDS. Blood.

[72] Gu Z (2014). Wnt/beta-catenin signaling mediates the senescence of bone marrow-mesenchymal stem cells from systemic lupus erythematosus patients through the p53/p21 pathway. Mol. Cell Biochem..

[73] Zhang DY (2013). Wnt/beta-catenin signaling induces the aging of mesenchymal stem cells through promoting the ROS production. Mol. Cell Biochem..

[74] Zhang DY, Wang HJ, Tan YZ (2011). Wnt/beta-catenin signaling induces the aging of mesenchymal stem cells through the DNA damage response and the p53/p21 pathway. PLoS ONE.

